# Health-Related Quality of Life in Previous Versus Current Opiate Users Receiving HCV Therapy: Registry-Based Evidence

**DOI:** 10.3390/brainsci16040414

**Published:** 2026-04-15

**Authors:** Michael Specka, Stefan Christensen, Peter Buggisch, Renate Heyne, Uwe Naumann, Hartwig Klinker, Ralph Link, Christiane Sybille Schmidt, Bernd Schulte, Jens Reimer, Fabrizio Schifano, Heiner Wedemeyer, Norbert Scherbaum

**Affiliations:** 1 Department of Psychiatry and Psychotherapy, LVR-University Hospital Essen, Faculty of Medicine, University of Duisburg-Essen, 45147 Essen, Germany; michael.specka@uni-due.de; 2 CIM Münster, 48143 Münster, Germany; christensen@cim-ms.de; 3 Department of Internal Medicine B: Gastroenterology, Hepatology, Endocrinology and Infectious Diseases, University Hospital Münster, 48149 Münster, Germany; 4 ifi-Institute for Interdisciplinary Medicine, 20099 Hamburg, Germany; buggisch@ifi-medizin.de; 5 Leberzentrum am Checkpoint, 10961 Berlin, Germany; heyne@leberzentrum-checkpoint.de; 6 UBN/Praxis, 14059 Berlin, Germany; naumann@ubn-praxis.de; 7 Division of Infectious Disease, Department of Internal Medicine II, University Hospital Würzburg, 97080 Würzburg, Germany; klinker_h@ukw.de; 8 MVZ-Offenburg GmbH/St. Josefs-Klinik, 77654 Offenburg, Germany; ralph.link@mvz-offenburg.de; 9 Center for Interdisciplinary Addiction Research (ZIS), Department of Psychiatry and Psychotherapy, University Medical Center Hamburg-Eppendorf (UKE), 20246 Hamburg, Germany; c.schmidt@zis-hamburg.de (C.S.S.); b.schulte@uke.de (B.S.); reimer@uke.de (J.R.); 10 Center for Psychosocial Medicine, Academic Teaching Hospital Itzehoe, 25524 Itzehoe, Germany; 11 Psychopharmacology, Drug Misuse and Novel Psychoactive Substances Research Unit, University of Hertfordshire Medical School, College Lane Campus Hatfield, Hatfield AL10 9AB, UK; f.schifano@herts.ac.uk; 12 Department of Gastroenterology, Hepatology, Infectious Diseases and Endocrinology, Hannover Medical School, 30625 Hannover, Germany; wedemeyer.heiner@mh-hannover.de; 13 Leberstiftungs-GmbH Deutschland, 30625 Hannover, Germany; 14 Center for Translational and Behavioral Neuroscience (CTNBS), University Hospital Essen, 45147 Essen, Germany

**Keywords:** opiate dependence, opiate abstinence, previous opiate addicts, health related quality of life, hepatitis C treatment

## Abstract

**Background**: Health and social outcomes of previous opiate users (POUs) are not well-documented. We characterize the life situation, health status, and health-related quality of life (HRQoL) of POUs entering antiviral hepatitis C (HCV) treatment, compared with HCV patients without past illicit opiate use (NOU), and with HCV patients currently in opiate agonist treatment (OAT). **Methods**: Data are taken from the German Hepatitis C-Registry (“Deutsches Hepatitis C-Register”, DHCR), a multi-centre registry study focussing on the course and outcome of HCV treatment with directly acting antivirals. At treatment entry, patients underwent a standardized clinical assessment, including the Short Form 36 (SF-36) for self-reported HRQoL. **Results**: POUs (*n* = 734) and OAT patients (*n* = 554) were similar with regard to age, sex, migrant background, and psychiatric comorbidity. Employment rate and cannabis, alcohol, and smoking abstinence rates were higher for POUs than for OAT patients, but still lower than for NOU (*n* = 4147) patients. Mental and physical HRQoL was better for POUs than for OAT patients, but worse than for NOU patients. Compared with SF-36 normative data, POUs showed decreased HRQoL, especially regarding mental health. **Conclusions**: Compared with opiate-dependent patients in OAT, POUs showed less psychotropic substance use and better HRQoL. Compared with NOU patients and the general population, mental health problems were especially increased. Challenges persist for POUs even during abstinence from opiates, highlighting the need for targeted interventions tailored to the specific needs of this population.

## 1. Introduction

Opiate dependence is a chronic, relapsing disorder. Both individuals in treatment and those outside of treatment experience a high burden of mental and somatic morbidity [1,2,3]. In Germany, illicit opiate (heroin) use is often preceded by non-opioid substance use. In addition, mental health problems and social disadvantages are common risk factors for heroin use [2]. In contrast to other countries, especially the USA, there was no opioid epidemic in Germany up to now. Nevertheless, individuals undergoing medical treatment for chronic pain with opioid analgesics in some cases develop prescription opiate use disorder or start nonmedical use of prescription opioids, either because of trying to alleviate pain or for psychotropic effects [4]. In Germany, the sociodemographic profiles of illicit opiate users and patients in pain treatment with opioids differ markedly, e.g., about 75% of illicit opiate users are male compared with 25% of those receiving opioid pain medication, and the mean age is about 44 years versus 72 years [5]. Also, most people with nonmedical use of prescription opioids do not go on to use heroin or other illegal opioids [6], and opiate dependants in Germany with illicit opiate use rarely report previous regular prescription opiate use [7].

Only a minority of affected individuals achieve abstinence from opiates and opiate maintenance therapies [8,9]. However, systematic research on the physical health and health-related quality of life of former opiate-dependent individuals remains sparse.

One important context in which previous opiate users (POUs) are encountered is the treatment of chronic hepatitis C virus (HCV) infection. HCV prevalence among illicit opiate users is very high, primarily due to sharing non-sterile injection equipment [10,11,12]. Globally, approximately half of people who inject drugs have been exposed to HCV [6]. In Germany, seroprevalence and HCV RNA rates among people who inject drugs have been reported as high as 75% and 54%, respectively [13], while slightly lower, yet substantial, RNA prevalence has been observed among individuals in OAT [14,15]. Data specific to POUs are limited, but available evidence from Israel suggests that they also exhibit high rates of HCV infection [16]. Beyond being a major cause of chronic liver disease, chronic HCV infection is associated with multiple extra-hepatic manifestations, including cardiovascular events, chronic kidney or end-stage renal disease, type 2 diabetes and insulin resistance, rheumatoid-like arthritis, neurocognitive dysfunction, and depression [17,18].

Although most of those affected by hepatitis C are asymptomatic [19], there is robust evidence that the hepatic and the extra-hepatic manifestations of a chronic HCV infection have a detrimental impact on health-related quality of life (HRQoL). Even the awareness of an HCV infection can lead to a reduced HRQoL [20], which further deteriorates with the progression of the liver disease and associated complications such as fibrosis, cirrhosis, hepatocellular carcinoma and/or hepatic encephalopathy or in presence of depressive and neuropsychiatric symptoms such as fatigue and cognitive impairments [21,22].

When compared with the general population, or with people with various medical illnesses, opiate dependent patients in OAT report lower HRQoL levels [23,24]. In addition, compared to HCV-negative OAT patients [24,25], and particularly so regarding mental health, OAT patients with chronic HCV infections reported moderately increased psychopathological burden and poorer HRQoL levels. In contrast, little is known about health and HRQoL of POUs with an HCV infection.

Using data from a nationwide German registry of patients treated for HCV, the present study aimed to describe the characteristics of previous opiate users at the start of HCV treatment, focusing on their health-related quality of life. Comparisons were made with the normative general population, patients currently in opiate agonist treatment, and patients without a history of opiate dependence, considering sociodemographic variables and comorbid conditions. Particular attention was given to analyzing differences in HRQoL across these groups.

## 2. Materials and Methods

### 2.1. Design

The German Hepatitis C-Registry (“Deutsches Hepatitis C-Register”, DHC-R) is a multi-centre, non-interventional registry study focusing on the course and outcome of hepatitis C treatment (HCT), with direct acting antivirals (DAA) medications offered at 160 centres throughout Germany at the time of the present analysis. Patient enrolment started in November 2014 and ended in March 2022.

Inclusion criteria for the DHC-R were adult age (≥18 years of age) and chronic hepatitis C, defined as detectable hepatitis C virus RNA. Main exclusion criteria were contraindications for DAA treatment and pregnancy of the patient. Before enrolment, patients had to provide written informed consent.

A proportion of DHC-R patients were opiate dependent and received opiate agonist treatment (OAT). In Germany, OAT is initiated and maintained mainly in specialized private practices and psychiatric outpatient clinics of hospitals, i.e., usually not in HCV-treatment facilities. In contrast, most HCV treatments are carried out by gastroenterologists and by infectious disease specialists. Costs for both treatments are fully covered by the mandatory general health insurance.

Included into the present study were patients with a history of illicit opiate use who were currently not using opiates (previous opiate user; POU); patients with no history of opiate dependence (NOU); and patients undergoing OAT. Patients with two HCV genotypes were excluded from the present analysis.

### 2.2. Assessments

At treatment entry, patients underwent a standardized assessment, which included hepatitis-related clinical and laboratory parameters, and recording of physical and psychiatric comorbidities. Demographic and clinical characteristics assessed upon enrolment included: age, sex, body mass index, prior HCV treatment experience, country of birth, self-reported somatic and psychiatric comorbidities and alcohol, tobacco and cannabis use. To estimate the liver fibrosis and cirrhosis severity, the AST-to-Platelet-Ratio-Index (APRI score) was calculated.

At treatment entry, the HRQoL Short Form 36 (SF-36) was administered, which is a self-administered questionnaire aiming at providing a comprehensive assessment of physical, mental, and social components of patients’ perceived health status. This tool has been translated into more than 40 languages and has been used for monitoring health the in context of a wide range of medical conditions [26]. The instrument includes 36 items to assess general health in eight domains (physical functioning, role limitations–physical, bodily pain, general health, vitality, social functioning, role limitations–emotional and mental health), which can be summarized into two comprehensive scores, the Physical (PCS) and the Mental Component Score (MCS). Higher scores mean better health functioning.

Patients’ scores in the eight domains were compared to normative data from the general population in Germany [27]. In the cited survey, data from 6964 participants were collected, and values for the first, second and third quartiles were reported, stratified by male/female sex and age group (18–29, 30–39, etc., up to age 79).

Patient data were collected by a web browser-based Electronic Data Capture (EDC) system without software installation on site (BEO, e.factum GmbH) hosted at a Clinical Research Organization (CRO). Data had been checked for plausibility as well as on site and remote monitoring.

### 2.3. Statistical Analyses

Pairwise comparisons between the three groups were performed using Chi^2^ tests of association for categorical variables, and Welch-corrected *t*-tests for interval-scaled variables. For each domain of the SF-36, the score of a patient was compared with normative data for his/her sex and age category. Descriptive statistics comprised the proportion of patients with scores within the lowest quartile of the general population. For SF-36 sum scores, one-sample *t*-tests were performed, comparing group means with the means of the normative sample. Given the large number of statistical comparisons, the level for rejecting a null hypothesis was set at *p* < 0.01, in order to reduce the risk of false-positive results.

## 3. Results

### 3.1. Sample Description

Patients initiated DAA treatment between February 2014 and November 2019. A total of 14,161 patients met the inclusion criteria, of whom 5435 were treated at facilities where the SF-36 health survey was documented. Out of 5435 patients, 734 patients had an opiate use history but no current use (previous opiate user, POU), 554 patients were currently in opiate agonist treatment (OAT), and 4147 patients had no history of illicit opiate use (no opiate use, NOU) at baseline.

The sociodemographic characteristics of each group are displayed in Table 1. Typical POU were males in their mid-40s (range 18–79 years). One fourth of the sample was either born abroad or had a first language other than German. Both groups with an opiate use history were markedly younger, had a much larger proportion of males and a lower proportion with migrant background than the NOU group. Employment rates of POUs were markedly higher than in OAT patients and similar to those identified in the NOU group.

With regard to hepatitis C-related indicators (Table 2), the cirrhosis rate in POUs was similar to NOU patients, and the proportion with low APRI score among POUs was higher than in the OAT and NOU groups (48.9% vs. 42.2% vs. 41.1%). Of particular importance were psychiatric diseases (depression or psychosis), which affected nearly one fourth of POUs. The rate for POUs was comparable with OAT patients, and much higher than in NOU patients. Regarding somatic diseases, nearly one in five POUs had a heart and/or circulatory disease—a significantly lower rate than in NOU patients, but higher than in OAT.

The rate for alcohol abuse or dependence among POUs was 17.4%, an increase in excess of five times in comparison with the NOU group, and higher than in OAT patients. The rate of tobacco smokers among POUs (73.3%) and OAT patients (83.2%) was much higher than in the NOU group (31.6%). In the POU group, 5.6% reported weekly cannabis use—a much lower proportion than in the OAT group (16.6%), but markedly higher than in the NOU group (1.3%).

### 3.2. Health-Related Quality of Life

**Figure 1:**
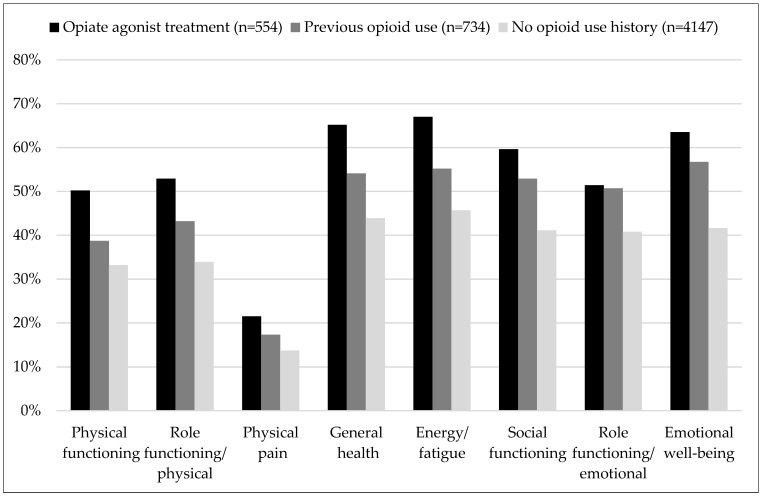
Proportions of patients with low health-related quality of life (within the lowest quartile of the general population, adjusted for age and sex) in the respective domains of the SF-36. In all domains except physical pain, previous opiate users (POUs) showed statistically significantly higher rates of low scores than the never users. In contrast, POUs showed less impairment than patients in opiate agonist treatment (OAT) in the domains “physical functioning”, “physical role functioning”, “general health” and “vitality”. Differences between POUs and OAT patients regarding “physical pain”, “social functioning”, “emotional functioning” and “emotional well-being” were not statistically significant.

For each patient, it was determined whether his/her SF-36 domain scores were within the lowest quartile of the reference population, stratified by sex and age. More than half of POUs showed low HRQoL in the domains of general health, energy/fatigue, social functioning, emotional role functioning, and emotional wellbeing (Figure 1). In addition, low physical functioning and physical role functioning were identified in about 40% of the POU sample. Only with regard to high levels of physical pain, the rate for POU was similar to the normative general population. Again, profiles of all three groups were very similar, with the NOU group on a more favourable level than POUs, and the OAT group showing worse values than the two other groups. Group comparisons between POUs and NOU patients resulted in *p* < 0.01 throughout, except “physical pain” (*p* = 0.011). Comparisons between the POU and OAT groups were statistically significant at the 0.01 level for “physical functioning”, “physical role functioning”, “general health” and “vitality”, but not for “physical pain” (*p* = 0.059), “social functioning” (*p* = 0.016), “emotional functioning” (*p* = 0.71) and “emotional well-being” (*p* = 0.012).

**Figure 2:**
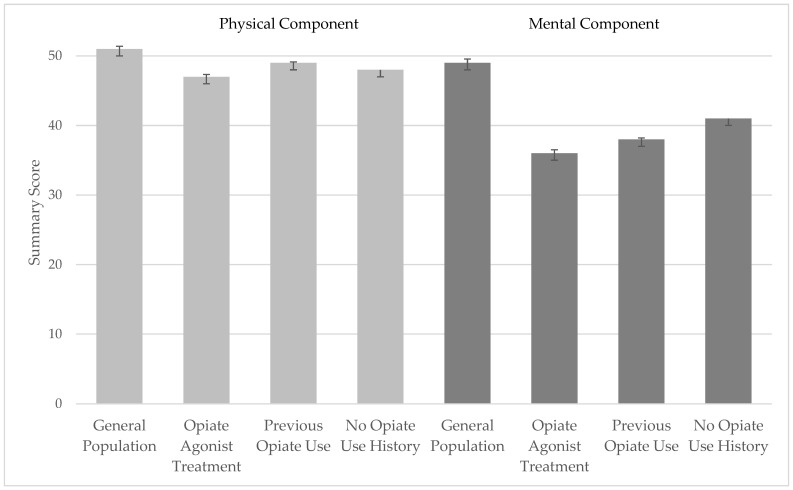
Means and 95% confidence intervals for the Physical Component Score and the Mental Component Score in the three patient groups and in the general population. Higher scores indicate better health related quality of life (HRQoL). Physical HRQoL was slightly decreased in all three patient groups, compared with the normative group. Mental HRQoL was markedly decreased, especially in patients currently undergoing opiate agonist treatment.

With regard to the SF-36 summary scores (Figure 2), the mean mental component scores were markedly lower in all three groups compared with a representative sample from the general population, which had a mean of 49.3 points [28]. In contrast, means for the physical component scores were only slightly lower than in the general population (e.g., 51.4 points). The POU group had the highest mean score among all groups for the physical component. For the mental component, the scores followed the order: NOU > POU > OAT. All pairwise comparisons were statistically significant (*p* < 0.01).

### 3.3. Representativeness of the Included Patients for the Whole Sample

Within the three patient groups, we compared patients with versus without HRQoL measurement, with respect to basic sociodemographic characteristics, mental health, liver damage, and substance use. In all three groups, mean age differed by 1 year or less between those with versus without HRQoL measurement. Percentage differences were smaller than 4% throughout for: proportion of males; being foreign-born; first language other than German; cirrhosis; APRI score > 1.5; current depression or psychosis; alcohol abuse; and cannabis consumption. Employment rates between subgroups were similar within OAT and NOU, but not within the POU subgroup (42.4% for included patients versus 32.2% for non-included patients). Strikingly, the rate of documented tobacco smokers was markedly increased for included patients, compared to those not included (POU: 73.7% vs. 49.6%; OAT: 83.2% versus 62.0%; NOU: 31.6% versus 24.7%).

## 4. Discussion

The present study characterized health status and health-related quality of life (HRQoL) levels of a large sample of previous opiate users (POUs) at the beginning of an antiviral treatment for hepatitis C in routine settings. POUs were predominantly males, with a median age of 47 years. About half of them were employed at the time of enrolment. Significant minorities showed psychiatric diseases and alcohol use disorder, and the vast majority were smokers. Compared with patients who had never used opiates (NOU), the POU group was markedly younger, with a higher proportion of males, and a lower proportion of individuals with migrant background. Compared with OAT patients, POUs were similar with regard to these characteristics. In contrast, employment rates for POUs were similar to NOU, and much higher than for OAT patients. It must be taken into account, though, that 90% of POUs were younger than 60 years, the typical working age, while this proportion was only 66% in the NOU group; therefore, it can be assumed that, in the NOU group, a considerable proportion of patients without employment had already retired, while in the POU group, they were in fact unemployed.

In the POU group, rates for cannabis use and for mental disorders were higher than in NOU, but lower than in OAT patients. However, alcohol abuse was markedly higher in the POU group (17.4%) than in the OAT group (12.1%) and in the NOU group (2.9%).

Regarding the domains of the SF-36, a large proportion and often the majority of POUs showed limited or poor health-related quality of life, except physical pain. The HRQoL profile of POUs was very similar to OAT and NOU groups, but the level of impairment was between these two comparison groups. In addition, POUs’ mean aggregate score for mental HRQoL was much lower than for the general population; the aggregate physical health score, in contrast, was not markedly different from the general population. Moreover, POUs showed significantly lower mental health scores than the NOU patients, but higher and statistically significant mental and physical health scores than OAT patients.

Health-related quality of life (HRQoL) is a multidimensional concept assessing how physical, mental, emotional, and social health affects an individual’s daily functioning and well-being. It captures a patient’s own perspective on how health conditions, treatments, or diseases impact their life, covering aspects like mobility, pain, and psychological state [29]. A number of factors influence HRQoL in individuals with HCV and in those with present or past opiate use.

Regarding physical factors, patients with chronic hepatitis C experience a variety of symptoms including fatigue, muscle aches, pruritis, swelling, sexual dysfunction, and nausea, all of which negatively affect HRQoL [30]. Moreover, the severity of the disease itself might directly influence HRQoL, although evidence is inconclusive [31]. Of particular concern here is the impact of chronic liver diseases on brain function via the liver-brain axis: Possible mechanisms are neuroinflammation due to hepatitis C as a chronic systemic inflammation with increased levels of peripheral cytokines, and gut dysbiosis leading to dysfunction of the intestinal barrier, which in turn results in systemic inflammation with increased cytokine levels and activated immune cells. In addition, there may be a malfunction of the afferent vagal nerve [32,33]. These mechanisms could lead to depressive symptoms, fatigue, and cognitive slowing [32]. Physical determinants based on a chronic hepatitis C infection affect all three groups included in the present study and contribute to their decreased physical and mental HRQoL, compared with the general population. Moreover, the knowledge of HCV positive status impacts HRQoL in addition to actually being infected with the virus. It is commonly reported that the HCV diagnosis elicits depressive symptoms; however, in active opiate users, this reaction is weak [34].

Regarding mental health factors, substance users in general show substantially increased rates of psychiatric disorders [2], which in turn decrease HRQoL. In the present study, the rate for depression and psychosis was markedly higher in the OAT group, and also in the POU group, than in the NOU group (which itself showed a rate twice as high as in the adult population in Germany [35]). Some mental health problems not associated with HCV could have persisted after chronic opiate use. This would contribute to the decreased mental component score in POUs compared with NOU patients.

Behavioural factors such as smoking and regular alcohol consumption were here more prevalent in OAT patients and POUs than in NOU patients. In general, active substance users show lower HRQoL than population norms, irrespective of their HCV status [34]. Finally, social factors such as discrimination, stigma, isolation are common among individuals with HCV [36], and even more so in active opiate users and those in OAT [37]. This might contribute to the decreased HRQoL in all groups and particularly in the OAT group. As indicated by the employment rate, the POU group appears less affected by social instability than the OAT group.

The high mental and somatic health burden of opiate dependent patients may remain even after years in OAT [38]. Chronic HCV infection, in particular, impairs the HRQoL of affected individuals through manifestations of the disease or certain symptoms such as fatigue, weakness and nausea and due to the substantial psychological impact of the infection [39]. The present findings, referring to a natural sample of OAT patients, are in line with observations from patients entering clinical trials of HCV treatment, where HRQoL scores of OAT patients were significantly lower than scores for the general population and lower than scores reported for other HCV patients [40]. The novel finding here relates to previous opiate users, who showed compromised HRQoL in nearly all domains considered, and who roughly fell between OAT patients and patients without an opiate use history.

The implications of this pattern of results are not immediately clear. In terms of mental health, the self-reported impairments in the POU group may reflect a combination of factors: pre-existing vulnerabilities associated with the initiation and maintenance of opiate dependence, the effects of previous opiate use itself, and the impact of hepatitis C infection. Previous research on the health of POUs has focused primarily on psychological variables and psychopathology. Compared with controls without a history of opiate use or with normative population values, POUs exhibit an increased mental health burden, particularly related to depression and anxiety [41,42,43]. There were also here some indications of high rates of chronic pain [16], and/or physical disability, both interfering with finding a job [9], and of alcohol/tobacco consumption [9,42]. Finally, POUs exhibited signs of premature ageing across various biological indicators of ageing [42].

DAA treatment in previous or current drug users, whether or not they are receiving opiate agonist therapy, can achieve sustained virological response rates comparable to those observed in patients without a history of drug use [44]. DAA treatment can also substantially improve patients’ quality of life, starting from the initial phases [39,45]. Regarding HRQoL, several studies indicate that DAA treatment leads to improvements [46,47], although improvements might only be modest [48,49] and may not be persistent in the long-term [50]. In a previous study with patients from the DHC-R, roughly half of all patients did not achieve a clinically important improvement (e.g., 2.5 points) in MCS and PCS. Low MCS and PCS scores were identified as predictors of achieving clinically meaningful improvement, with patients who have lower baseline HRQoL appearing to benefit the most from modern therapeutic options [20]. Both POUs and OAT patients were here clearly identified as those patient groups more in need of improving their HRQoL, hence most possibly taking advantage from antiviral treatment in this respect.

In particular, the POU group showed issues regarding mental health, cannabis and alcohol use. Besides the high risk of a relapse into a dependent opiate use condition, these problems may well need to be therapeutically addressed in order to increase the quality of life of POUs. However, there is concern that this group may face difficulties in accessing adequate treatment, as the standard approach for opiate-dependent individuals is OAT. The problem burden of the POU group fell between that of the NOU and OAT groups, suggesting that a minority of opiate-dependent individuals may achieve abstinence, which could in turn improve their quality of life, for example through better professional integration. Conversely, individuals with opiate dependence who have a lower level of associated problems than the average OAT patient may find it easier to achieve and maintain abstinence.

### Limitations

A number of limitations have to be considered here. Information about somatic and mental comorbidities and the use of psychoactive substances was based on self-reports. Such data may present with low levels of reliability, due to both recollection problems and incorrect assumptions about existing diagnoses and can be biassed as well (e.g., by underreporting of levels of substance use). These data were not validated by other information sources such as drug testing or previous medical files.

The present POU sample included individuals with hepatitis C who had used opiates in the past, most typically through IV injection. It is not immediately clear here whether results can be generalized to non-infected or to previously non-IV-using groups.

Moreover, opiate abuse history and treatment history were here not documented. One major problem resulting from this is the unknown degree of heterogeneity within the POU group. It is plausible to assume that the POU group consists of patients with short, medium-term, or long-term abstinence, each exhibiting different patterns of medical and social problems and different HRQoL. The long-term course of illicit opiate dependence typically comprises recurring sequences of addictive opiate use, abstinence, and OAT, therefore stable recovery from drug dependence cannot safely be assumed especially for those patients with shorter duration of abstinence [8].

Another concern could be a possible bias in the results, as the HRQoL questionnaire was not applied in all eligible patients. However, sociodemographic, health related, and substance use characteristics were very similar between patients included versus not included in the present study, with the exception of an increased employment rate of included patients of the POU group, and much higher smoking rates in all three included groups. For both observations we can offer no immediate explanation. The comparatively lower smoking rate could imply that the health-related quality of life would be better in those patients not included.

Finally, the instrument for measurement of HRQoL, the SF-36, was not specifically developed for, or adapted to, populations with an HCV infection. Indeed, some items or subscales from the instrument might possess low discriminatory power, especially when used with younger patients. The normative values for the respective domains were collected from the German general population in the late 1990s and may have changed since then, although the direction and magnitude of any such shifts are unknown.

## 5. Conclusions

Previous opiate-dependent individuals who are currently abstinent exhibit lower HRQoL compared with those who have never used opiates, particularly in the mental health domain. Patients currently receiving opiate agonist treatment demonstrate an even lower HRQoL than both abstinent former users and individuals with no history of opiate use.

A possible focus of future research is the extent of improvement of HRQoL of previous opiate users during DAA treatment, and whether additional interventions for this group, especially regarding mental health and substance-related disorders, might improve the quality of life of this group.

## Figures and Tables

**Table 1:** Sociodemographic patient characteristics.

	Previous Opiate User (POU) *n* = 734	Opiate Agonist Treatment (OAT) *n* = 554	No Opiate Use (NOU) *n* = 4147	Pairwise Comparisons ^(1)^, *p* Values
Age				
Mean (SD)	46.4 (10.0)	44.6 (9.0)	53.3 (13.0)	POU vs. NOU: <0.0001 POU vs. OAT: 0.025 OAT vs. NOU: <0.0001
Min-Max/Median	18–79/47	22–82/45	18–87/54	
Age ≥ 60 years	9.4% (*n* = 69)	4.6% (*n* = 25)	32.7% (*n* = 569)	
Male sex	74.4% (*n* = 546)	79.8% (*n* = 442)	56.2% (*n* = 2330)	POU vs. NOU: <0.0001 POU vs. OAT: 0.02 OAT vs. NOU: <0.0001
Foreign born	26.3% (*n* = 193)	28.2% (*n* = 156)	35.8% (*n* = 1484)	POU vs. NOU: <0.0001 POU vs. OAT: 0.46 OAT vs. NOU: <0.0001
First language not German	25.5% (*n* = 187)	27.3% (*n* = 151)	34.5% (*n* = 1432)	POU vs. NOU: <0.0001 POU vs. OAT: 0.47 OAT vs. NOU: <0.0001
Employed at enrolment	42.4% (*n* = 311)	27.3% (*n* = 151)	45.6% (*n* = 1892)	POU vs. NOU: <0.0001 POU vs. OAT: <0.0001 OAT vs. NOU: <0.0001

^(1)^ Chi square tests or independent samples *t*-test with Welch correction, respectively.

**Table 2:** Hepatitis and other health-related characteristics.

	Previous Opiate User (POU) *n* = 734	Opiate Agonist Treatment (OAT) *n* = 554	No Opiate Use (NOU) *n* = 4147	Pairwise Comparisons ^(1)^, *p* Values
Previous HCV therapies	24.8% (*n* = 182)	17.7% (*n* = 98)	31.8% (*n* = 1317)	POU vs. NOU: <0.001 POU vs. OAT: 0.09 OAT vs. NOU: <0.0001
Cirrhosis	20.3% (*n* = 149)	16.6% (*n* = 92)	21.6% (*n* = 894)	POU vs. NOU: 0.44 POU vs. OAT: 0.09 OAT vs. NOU: <0.01
APRI-Score				
≤0.5 (probably no fibrosis)	48.9% (*n* = 336)	42.2% (*n* = 217)	41.1% (*n* = 1506)	POU vs. NOU: <0.001 POU vs. OAT: 0.02 OAT vs. NOU: 0.63
>0.5–≤1.5 (undecided)	36.1% (*n* = 248)	39.5 (*n* = 203)	41.5% (*n* = 1521)	POU vs. NOU: 0.01 POU vs. OAT: 0.25 OAT vs. NOU: 0.38
>1.5 (probable fibrosis)	15.0% (*n* = 103)	18.3% (*n* = 94)	17.4% (*n* = 637)	POU vs. NOU: 0.13 POU vs. OAT: 0.13 OAT vs. NOU: 0.61
Current depression or psychosis	23.0% (*n* = 169)	25.1% (*n* = 139)	14.1% (*n* = 584)	POU vs. NOU: <0.0001 POU vs. OAT: 0.39 OAT vs. NOU: <0.0001
Active smoker	73.7% (*n* = 541)	83.2% (*n* = 461)	31.6% (*n* = 1312)	POU vs. NOU: <0.0001 POU vs. OAT: <0.0001 OAT vs. NOU: <0.0001
Alcohol abuse/dependence	17.4% (*n* = 128)	12.1% (*n* = 68)	2.9% (*n* = 119)	POU vs. NOU: <0.0001 POU vs. OAT: 0.01 OAT vs. NOU: <0.0001
Current alcohol consumption				
>40 g/d (men) or >30 g/d (women)	4.9% (*n* = 36)	7.2% (*n* = 40)	2.0% (*n* = 85)	POU vs. NOU: <0.0001 POU vs. OAT: 0.08 OAT vs. NOU: <0.0001
No consumption	74.5% (*n* = 547)	69.3% (*n* = 384)	79.2% (*n* = 3285)	POU vs. NOU: <0.01 POU vs. OAT: 0.04 OAT vs. NOU: <0.0001
Cannabis consumption at least weekly	5.6% (*n* = 41)	16.6% (*n* = 92)	1.3% (*n* = 54)	POU vs. NOU: <0.0001 POU vs. OAT: <0.0001 OAT vs. NOU: <0.0001

^(1)^ Chi square tests or independent samples *t*-test with Welch correction, respectively.

## Data Availability

Access to individual data sets is not available due to informed consent. Upon request, aggregated data can be made available.

## Abbreviations

The following abbreviations are used in this manuscript:

APRI	AST-to-platelet-ratio-index
AST	aspartate aminotransferase
CRO	clinical research organization
DAA	directly acting antivirals
DHCR	Deutsches Hepatitis C Register (German hepatitis C registry)
EDC	electronic data capture
HCV	hepatitis C virus
HRQoL	health-related quality of life
MCS	mental component score
NOU	no opiate use
OAT	opiate agonist treatment
PCS	physical component score
POU	previous opiate user
SF-36	Short Form 36
